# Effectiveness of treatment for 6813 patients with mental health conditions in Cambridgeshire: a cross-sectional study

**DOI:** 10.1192/bjo.2020.14

**Published:** 2020-03-20

**Authors:** Nathan J. Dean, Nikitas Arnaoutoglou, Benjamin R. Underwood

**Affiliations:** Clinical School of Medicine, Cambridge University, UK; Windsor Research Unit, Fulbourn Hospital, UK

**Keywords:** Mental health, health of the nation outcomes scales, outcome measure, routine data

## Abstract

**Background:**

The Health of the Nation Outcomes Scales (HoNOS) has been widely used as an outcome measure in UK mental health settings for the past decade. The data-set gathered provides a unique opportunity to evaluate the effectiveness of the totality of mental healthcare in ‘real-world’ conditions; much of our clinical evidence currently comes from highly parameterised clinical trials investigating single interventions in highly selected patients.

**Aims:**

To examine all outcomes measured by HoNOS for a range of diagnostic groups, evaluate the influence of patient demographics on those outcomes, and observe changes in patient groups over time.

**Method:**

Here we show the data from 6813 adult patients treated in Cambridgeshire between 2012 and 2017. Patients were split into three diagnostic groups: psychosis, non-psychosis and organic. Changes in HoNOS scores from initial assessment to discharge were tested and regressions were used to evaluate the influence of age, gender and ethnicity on the changes, as well as to model changes in the severity of initial presenting symptoms with time.

**Results:**

HoNOS scores significantly improve after treatment for psychotic, non-psychotic and organic conditions in adults and older adults. Age, but not gender or ethnicity, influenced change in HoNOS scores. Patients entering secondary mental health services had increased initial HoNOS scores over time.

**Conclusions:**

The UK repository of HoNOS scores provides a significant and relatively underutilised resource that can be exploited to gain insights into mental illness and treatment effectiveness. This is likely to have many applications, including influencing the commissioning of services.

## Outcome measures and payment in mental health

The Health of the Nation Outcomes Scales (HoNOS) was developed by the Royal College of Psychiatrists during the early 1990s^1^ and consists of 12 scales measuring a patient's behaviour, impairment, mental health symptoms and social functioning. It was developed at least in part to provide an outcome measure for patients receiving routine mental healthcare spurred by the 1992 Health of the Nation strategy and its ambition ‘to improve significantly the health and social functioning of mentally ill people’.^2^ Since 2003, the Mental Health Minimum Data Set has made it mandatory for trusts across the UK to routinely assess the outcomes of patients accessing secondary mental health services,^3^ and trusts were encouraged to implement the use of HoNOS into their services.^4^ This change was meant to allow mental health trusts to move towards a system of payment by results rather than a block contract payment, and thus an equal financial footing and ‘parity of esteem’ with physical health trusts.^5^ Despite a significant amount of resource being spent on training, collecting and recording HoNOS data on a national scale, this change in funding model has yet to be realised in the UK. The resources expended will not be wasted if this data-set can be exploited for patient benefit, and using routine data to improve patient care is described in the UK National Health Service (NHS) constitution.^6^

## An alternate use for outcome measure data-sets

UK mental health data-sets provide a unique opportunity to evaluate the effectiveness of routine NHS care in mental health services. This care is evidence based, for example using the National Institute for Health and Care Excellence guidelines, and in turn these draw from high-quality studies of treatment efficacy. However, there is a known disparity between results from tightly parameterised randomised controlled trials and treatment outcomes in routine care (i.e. the distinction between efficacy and effectiveness). Evaluating the HoNOS data-set in the UK provides a unique insight into the effectiveness of modern mental healthcare, at scale. Previous work in Italy and Australia has been encouraging in looking at outcomes from generic mental health treatment,^7,8^ while in the UK, studies have examined predictors of outcome and variability between providers^9^ as well as outcomes for individual services,^10–14^ on the whole showing a picture of improved outcomes. In reality, patients often move between several services during the course of their treatment and thus what remains of interest, not least to patients, is whether outcomes improve from the experience as a whole.

## Aims

Here we present the analysis of nearly 7000 pairs of HoNOS scores for unique individual patients coming in to and leaving services in Cambridgeshire and Peterborough NHS Foundation trust (CPFT). Our aims are to look at all outcomes measured by HoNOS (symptom severity and social function) for a range of diagnostic groups and evaluate the influence of age, ethnicity and gender on those outcomes, as well as illustrate how data-sets of this sort can give insight into changes in patient groups over time and consider their implications for mental health services in the UK. Here we present, to our knowledge, the largest outcome measures data-set to examine the effectiveness of modern mental healthcare at scale in the UK.

## Method

### Ethical approval

This project was undertaken though and approved by the quality assurance and clinical effectiveness department at CPFT. It was undertaken as a service improvement project and met all trust governance requirements.

### CPFT

CPFT provides mental and community physical health services that are free at the point of access, to the County of Cambridgeshire, UK. The total population served is around 1 million and includes more than 165 000 people over the age of 65. The trust employs some 4000 staff who deliver around a million patient contacts annually, including more than 150 000 patient contacts to adults with mental health difficulties per year. CPFT's children's services provide more than 100 000 patient contacts per year. In this paper we do not investigate children's services directly as HoNOS is not universally used as an outcome measure in this service.^15^

### Data collection

Staff involved in the HoNOS scoring of patients all received a minimum of 4 h training via a workshop followed by trainer visits to individual teams, in order to minimise the potential variability in HoNOS scoring among clinicians.^16^ The original HoNOS (now HoNOS for working age adults) was used in all assessments. Since its inception, numerous adaptations of HoNOS have been made including specific versions for elderly people, children, individuals with intellectual disabilities (known in UK health services as learning disabilities), secure settings and for those with acquired brain injury. The use of a single scale here allows for uniformity and easy comparison across different services. Following a clinical assessment of the patient, clinicians would rate each of the 12 scales on a five-point system. All scales follow the same format^17^: 0, no problem; 1, minor problem requiring no action; 2, mild problem but definitely present; 3, moderately severe problem; 4, severe to very severe problem.

In addition to HoNOS scores on admission and discharge from CPFT services, information collected also included patient demographics (date of birth, gender, ethnicity). The information was stored in an anonymised database held and managed by the Trust's head of information and performance.

### Inclusion criteria

This retrospective study analyses the data-set of patients aged over 18 admitted and discharged from CPFT mental health services – not including the Improving Access to Psychological Therapies services – between January 2012 and December 2017. Only patients with at least two HoNOS scores, one on initial assessment and the one on discharge, were used for this study. We have only examined unique individuals and therefore the data-set does not include the same patient more than once.

### Statistical analyses

Patients were split into three diagnostic clusters derived from the Mental Health Clustering Tool (MHCT).^18^ The MHCT is derived from HoNOS and was developed in partnership between the Department of Health and the Royal College of Psychiatrists to group patients with similar needs and symptom severities into clusters:^18^

- non-psychosis – HoNOS cluster scores 1–8;
- psychosis – HoNOS cluster scores 10–17;
- organic – HoNOS cluster scores 18–21.

To test whether patients improved during their treatment in CPFT, changes in HoNOS scores between initial assessment and discharge were analysed using paired two-tailed *t*-tests. Within each of the three diagnostic clusters, patients were further split by age into <65 years old and ≥65 years old and the statistical tests were repeated. A *P* of <0.05 was considered statistically significant. The *P*-values presented are not corrected for multiple comparisons. In nearly all cases, doing so does not influence significance because of the large total population size, although we acknowledge the conceptual difficulties of relying on statistical inference in non-randomised data and use the *P*-values as a guide to interpretation.

To judge whether a patient's initial age at assessment had an impact on their mean change in total HoNOS scores, linear regression models were created for each patient diagnostic group, first across patients of all ages and then separately for patients aged <65 and ≥65 years.

A multiple ANOVA was used to test whether a patient's gender, ethnicity or gender × ethnicity had an impact on their mean change in total HoNOS scores from initial assessment to discharge.

To test whether there was any change in the severity of presenting symptoms between 2012 and 2017, a linear regression model was generated between the date of initial assessment and the mean initial scores across each of the different HoNOS subscales. Statistical analyses were performed using XLSTAT 2019 for Windows.

## Results

In total, 26 034 patients within the defined parameters entered CPFT mental health services, of which 16 658 (64.0%) had at least one HoNOS score collected and 6813 (26.2%) had at least two scores, one on initial assessment and one on discharge, and were thus eligible for this study. Table 1 shows the descriptive statistics for the sample population of patients in this study.

**Table 1:** Descriptive statistics

	Non-psychosis cluster	Psychosis cluster	Organic cluster
Number of patients in group	2592	495	3726
Percentage of patients in group	38.0	7.3	54.7
Age group, years			
<65	1556	361	146
≥65	1036	134	3580
Gender			
Male	852	247	1543
Female	1740	248	2183
Ethnicity			
White	1946	365	2742
Black	15	16	18
Asian	17	27	53
Mixed	17	13	41
Other	13	15	20
Unknown	584	59	852

### Change in HoNOS scores before and after treatment in CPFT

**Fig. 1:**
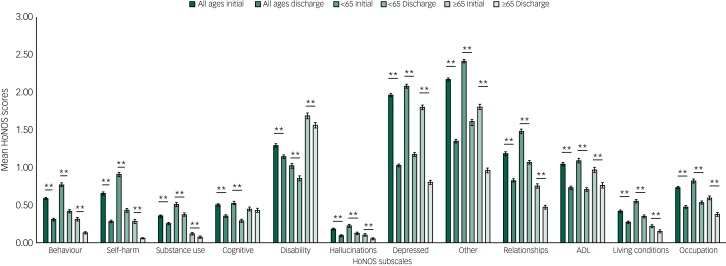
Non-psychosis disorder group: mean Health of the Nation Outcomes Scales (HoNOS) scores on initial assessment and discharge across the 12 HoNOS subscales.

Patients admitted with non-psychotic mental health conditions showed significant improvements across all 12 HoNOS subscales and in their total HoNOS score (mean initial score   11.07, mean discharge score 7.11, *P* < 0.001). Broken down by age, both the <65 and ≥65 groups showed significant improvements in all subscales with a mean initial score ≥1 (a score that signifies that the problem measured by the subscale is at a minimum a minor problem for that group of patients, and excludes the evaluation of subscales that are, on the whole, not problems for that group of patients). Across both age groups, the greatest improvements were in the ‘depression’ and ‘other mental and behavioural’ subscales, the two subscales that patients scored most highly on in the initial assessment (Fig. 1).

ADL, activities of daily living; s.e. bars are shown. *P < 0.05, **P < 0.001.

**Fig. 2:**
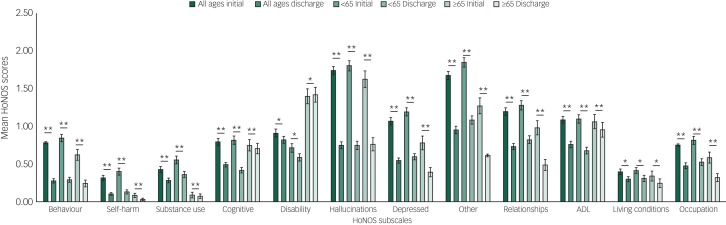
Psychosis disorder group: mean Health of the Nation Outcomes Scales (HoNOS) scores on initial assessment and discharge across the 12 HoNOS subscales.

Patients treated for psychotic mental illnesses also showed significant improvements in total HoNOS score (mean initial score  11.12, mean discharge score 6.48, *P* < 0.001) and across all 12 HoNOS subscales. Patients aged <65 show significant improvements across all 12 HoNOS subscales whereas patients aged ≥65 show improvements in all scales in which their initial scores were ≥1 except ‘disability’ and ‘activity of daily living’ (ADL) scales, which showed no significant improvements. Across both age groups, the most notable improvements were in the ‘hallucinations’ and ‘other mental and behavioural’ subscales (Fig. 2).

ADL, activities of daily living; s.e. bars are shown. *P < 0.05, **P < 0.001.

**Fig. 3:**
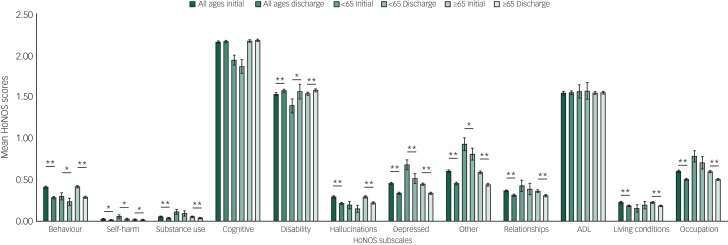
Organic disorder group: mean Health of the Nation Outcomes Scales (HoNOS) scores on initial assessment and discharge across the 12 HoNOS subscales.

Among patients with organic conditions there was a significant decrease in total HoNOS score at all ages (mean initial score 8.25, mean discharge score  7.63, *P* < 0.001). In both the <65 and ≥65 age groups, there were no significant improvements in any of the subscales that had a score of ≥1 on initial assessment: ‘cognitive’, ‘disability’ and ‘ADL’. For the group <65, the score for the ‘disability’ subscale increased during treatment (Fig. 3).

ADL, activities of daily living; s.e. bars are shown. *P < 0.05, **P < 0.001.

### Age and change in total HoNOS score

**Fig. 4:**
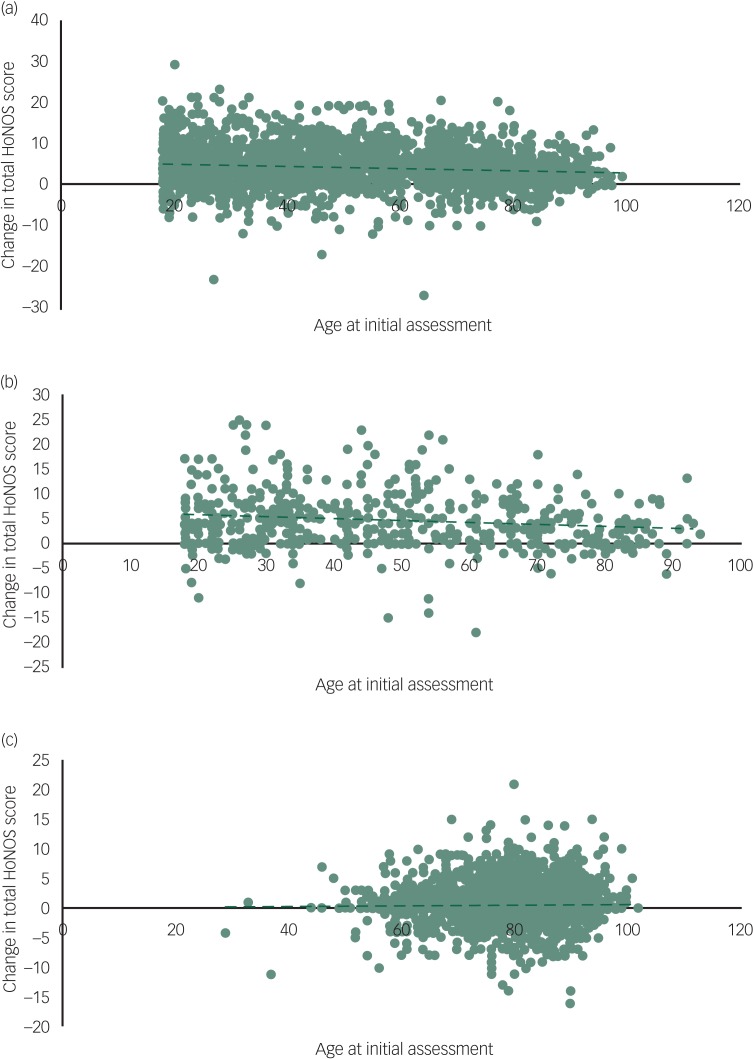
Age and change in total Health of the Nation Outcomes Scales (HoNOS): age of patients at initial assessment versus change in total HoNOS score for (a) non-psychosis, (b) psychosis and (c) organic clusters.

The linear regression model for the non-psychosis group across all ages shows a significant negative association between age and change in total HoNOS score, i.e. older patients have smaller changes in total score (*R*^2^ = 0.010, *F*(1,2673) = 27.5, *P* < 0.001) (Fig. 4(a)). When split into age groups, there was no significant association between age and change in HoNOS scores for patients aged <65 (*R*^2^ = 0.001, *F*(1,1637) = 1.65, *P* = 0.20) however, there was a significantly negative association for patients aged ≥65 (*R*^2^ = 0.013, *F*(1,1033) = 13.8, *P* < 0.001).

For the psychosis group, the linear regression model showed a significant negative association across all ages (*R*^2^ = 0.018, *F*(1,503) = 9.27, *P* < 0.01) (Fig. 4(b)), however, there was no significant association in the <65 age group (*R*^2^ < 0.001, *F*(1,369) = 0.080, *P* = 0.78), nor in the ≥65 group (*R*^2^ = 0.015, *F*(1,131) = 1.98, *P* = 0.16) when evaluated separately.

In the organic group, there was no significant association when modelled across all ages (*R*^2^ < 0.001, *F*(1,3723) = 0.37, *P* = 0.55) (Fig. 4(c)), nor in the <65 age group (*R*^2^ < 0.01, *F*(1,143) = 0.96, *P* = 0.33) or in the ≥65 age group (*R*^2^ < 0.001, *F*(1,3577) = 0.001, *P* = 0.98).

### Gender, ethnicity and change in total HoNOS score

For each of the patient groups, the multiple regressions model showed that there was no significant main effect of gender, ethnicity or gender × ethnicity (non-psychosis cluster: *F*(12,2579) = 1.20, *P* = 0.28; psychosis cluster: *F*(13, 481) = 1.18, *P* = 0.29; organic cluster: *F*(13,3712) = 0.70, *P* = 0.77).

### Severity of initial presenting symptoms of patients referred to CPFT secondary mental health services between 2012 and 2017

**Fig. 5:**
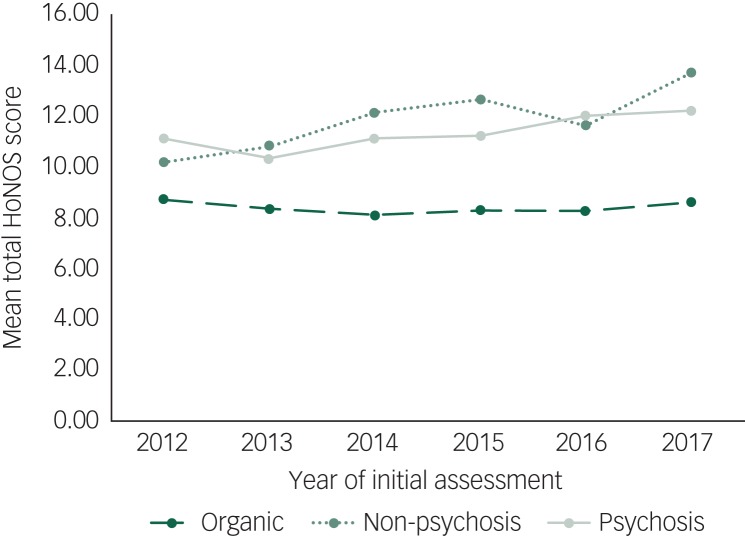
Mean total Health of the Nation Outcomes Scales (HoNOS) score at initial assessment against year of initial assessment for the three different diagnostic clusters.

Between 2012 and 2017, the mean total HoNOS score at initial assessment for patients presenting with non-psychotic conditions significantly increased (*R*^2^ = 0.004, *F*(1,2673) = 9.34, *P* < 0.01) indicating that the initial presenting problems for patients in these groups were on average more severe (Fig. 5). In this group of patients, initial scores for the ‘self-harm’, ‘depression’ and ‘other mental and behavioural’ subscales significantly increased whereas the initial score for the ‘relationships’ subscale significantly decrease.

For patients presenting with psychosis, the mean total HoNOS scores at initial assessment also increased between 2012 and 2017 (*R*^2^ = 0.024, *F*(1,503) = 12.1, *P* < 0.001) (Fig. 5). No individual subscales showed a significant reduction in initial scores. ‘Behavioural’, ‘cognitive’, ‘hallucinations’ and ‘other mental and behavioural’ subscales all showed a significant increase in initial scores during this period.

For patients with organic conditions, although the mean total HoNOS scores at initial assessment did not increase between 2012 and 2017 (*R*^2^ < 0.001, *F*(1,3723) = .29, *P* = 0.59) (Fig. 5), all the subscales with scores >1 at initial assessment – ‘cognitive’, ‘disability’ and ‘ADL’ – did significantly increase.

## Discussion

### Main findings

The mandate to collect treatment outcome measures using HoNOS in the UK has resulted in a large data-set of outcomes for patients receiving routine psychiatric care in the NHS. Here we show the data from nearly 7000 patients in Cambridgeshire treated over a 5-year period.

The data suggests that routine psychiatric practice in the UK is effective. Significant improvement is seen at all ages in psychotic and non-psychotic disorders, with the greatest improvement in the symptom domains with the greatest scores on entry into the services. These scores are recorded by staff and it is therefore possible, despite training and previous studies demonstrating the reliability of HoNOS, that these scores may not be accurate if staff are more optimistic on scoring symptoms at the end of treatment rather than the beginning. As such it is reassuring that symptom domains that would seem likely to be hard to treat or that are expected to deteriorate, for example cognition in progressive dementia, do not significantly improve. Despite this it was clear that total HoNOS scores for those with organic conditions still decreased across all ages, indicating that even the more elderly patients with dementias benefit from treatment they receive in secondary mental health services.

The relative efficacy of treatment for mental disorder across the age range remains an area of interest and is now being addressed by large randomised controlled trials specifically in older people.^19^ Our data moves beyond narrow efficacy to look at effectiveness of routine NHS care for older people with mental illness. We did see an effect of age on HoNOS scores in psychotic and non-psychotic mental illness, although only the latter result was true when examining only the patients aged over 65. It is possible that this represents the increased likelihood of mental illness being in the context of dementia in later life where we know treatment of other symptoms may be less effective. For example, antidepressants are not effective in treating depression in the context of Alzheimer's disease.^20^ Interestingly we saw no influence of age on outcomes in organic disorders, which does not support the idea that young-onset dementia differs to later-onset disease in terms of routine treatment outcomes.

Literature from the UK has previously raised concerns that experience of mental healthcare may be influenced by ethnicity.^21^ This was not reflected in our data where we saw no difference in terms of outcome by ethnicity or gender, although those from ethnic minorities formed a small part of the total sample, which is reflective of the ethnic make-up of the county as a whole.

Patients being referred into CPFT mental health services appear to be becoming more unwell on entry into the service as reflected by higher initial HoNOS scores with time. This may reflect increased pressure on NHS services because of increased demand. One outcome of this may be that the ‘threshold’ for entry to care is raised. This change is not seen in those with organic disease. This may be because many patients in this service are being referred for initial diagnostic assessment. Enthusiasm for early assessment and treatment in the UK may have resulted in patients being referred for diagnosis at less severe stages, which may more than counterbalance any increase in severity of patients being referred later in the disease course, for example to older age crisis teams. Overall the data does support the clinical concern that patients are increasingly unwell when referred.

The size of the data-set is a strength of this study and allows confidence in the significance of the results. However, the set of complete HoNOS pairings represents only a sample of the total patients receiving treatment in the Trust. This is not unique to the CPFT's data-set; incomplete HoNOS data-sets, where only a fraction of patients have had HoNOS scores recorded at least twice, has been a common finding among studies looking at HoNOS data-sets.^7–9,22^ Barriers to collecting routine outcome measurements may stem from clinicians being unable to see the way these improve their services or improve patient care, and this may be because of a lack of feedback mechanisms implemented across services or within trusts.^23^

### Limitations

Our study has several limitations. One should be cautious in inferring a causal relationship between receiving treatment from secondary mental health services and improving outcome measures based on our results. It seems likely that it was the treatment patients received that led to improvements as reflected in change in HoNOS score. However, we would need to analyse a control population of matched patients who did not receive any treatment over the same period to explore causality; a randomised trial on this scale is not possible, hence the value of routinely collected observational data. A limitation of large, observational data are that it may provide high power for small, insignificant effect sizes. Whether any particular change is clinically significant is open to debate. However, the overall improvements following treatment of psychotic and non-psychotic disorders are close to or exceed a four-point cut-off previously suggested for clinically significant change.^24^ The completeness, or lack thereof, of our data-set may also be a limitation of this study. Our data-set consists of only a fraction of all patients receiving treatment from CPFT mental health services and this leads to the possibility that our data-set represents a skewed patient sample. However, we have investigated which clinical teams provided data and found that there was good coverage of the different clinical services (supplementary Table 1 available at https://doi.org/10.1192/bjo.2020.14), thus we do not think the data are significantly affected by uneven distribution between clinical teams.

### Implications and future directions

The collection of large-scale data on outcomes in routine services allows accurate evaluation of outcomes for patients receiving routine care. Our results suggest that mental health services are effective in treating the symptoms and social problems with which patients present. Age has a small effect on the degree of improvements for adults with psychotic and non-psychotic illnesses, but not for organic illnesses. Importantly, gender and ethnicity do not induce differential outcomes. Patients appear to be presenting to services with more severe symptoms.

This data could be compared across trusts, or indeed collated at a national level. Although the original intention of using outcomes to influence funding for services now seems unlikely, the potential in this data for helping us understand mental illness and improve outcomes is significant. This data has already been collected at significant cost. Including training we estimate that the time required to create the data-set here is in excess of 10 000 h of clinician time. Nevertheless, these data exist and are available. As Wing et al said in their original paper describing HoNOS: ‘The key test for HoNOS is that clinicians should want to use it for their own purposes’.^1^

## Data Availability

The anonymised database used for this study is available from the corresponding author upon reasonable request.

## Supplementary material

For supplementary material accompanying this paper visit http://dx.doi.org/10.1192/bjo.2020.14. click here to view supplementary material

## References

[1] Wing JK, Curtis RH, Beevor AS. Health of the Nation Outcome Scales (HoNOS). Br J Psychiatry 1999; 174: 432–4. 10616611 10.1192/bjp.174.5.432

[2] Department of Health. Health of the Nation: A National Strategy for Health in England. HMSO, 1992.

[3] NHS Information Authority. The Mental Health Minimum Data Set. NHS, 2003 (https://www.datadictionary.nhs.uk/version2/data_dictionary/messages/mental_health_minimum_data_set/mental_health_minimum_data_set_definition_fr.asp?shownav=1 [cited 19 Aug 2019]).

[4] Fonagy P, Matthews PS. Outcomes Measures Implementation Best Practice Guidance. National Institute for Mental Health in England, 2005.

[5] NHS England. Developing a Capitated Payment Approach for Mental Health Detailed Guidance. NHS England, 2016.

[6] NHS Constitution. NHS Constitution – Interactive Version. NHS England, 2009 (https://www.nhs.uk/NHSEngland/aboutnhs/Documents/NHS_Constitution_interactive_9Mar09.pdf).

[7] Monzani E, Erlicher A, Lora A, Lovaglio P, Vittadini G. Does community care work? A model to evaluate the effectiveness of mental health services. Int J Ment Health Syst 2008; 2: 10. 18601741 10.1186/1752-4458-2-10 PMC2488329

[8] Burgess P, Pirkis J, Coombs T. Do adults in contact with Australia's public sector mental health services get better? Aust New Zealand Health Policy 2006; 30: 3–9. 10.1186/1743-8462-3-9 PMC1570132 16942623

[9] Moran V, Jacobs R. Comparing the performance of English mental health providers in achieving patient outcomes. Soc Sci Med 2015; 140: 127–35. Available from: 10.1016/j.socscimed.2015.07.009. 26218853

[10] Stephenson LA, Macdonald AJD, Seneviratne G, Waites F, Pawlby S. Mother and Baby Units matter: improved outcomes for both. BJPsych Open 2018; 4(3): 119–25. 29971155 10.1192/bjo.2018.7 PMC6020269

[11] Veerbeek MA, Voshaar RCO, Pot AM. Effectiveness and predictors of outcome in routine out-patient mental health care for older adults. Int Psychogeriatr 2014; 26(9): 1565–74. 10.1017/S1041610214000647 24758711

[12] Mujic F, Cairns R, Mak V, Squire C, Wells A, Al-harrasi A, Liaison psychiatry for older adults in the general hospital: service activity, development and outcomes. BJPsych Bulletin 2018; 42(1): 30–6. 29388526 10.1192/bjb.2017.9 PMC6001875

[13] Penno SJ, Hamilton B, Petrakis M. Early intervention in psychosis: Health of the Nation Outcome Scales (HoNOS) Outcomes from a five-year prospective study. Arch Psychiatr Nurs 2017; 31(6): 553–60. Available from: 10.1016/j.apnu.2017.07.003. 29179820

[14] Butt MF, Walls D, Bhattacharya R. Do patients get better? A review of outcomes from a crisis house and home treatment team partnership. BJPsych Bulletin 2019; 43(3): 106–11. 30693856 10.1192/bjb.2018.105 PMC8058930

[15] Care Quality Commission. Cambridgeshire and Peterborough NHS Foundation Trust: Inspection Report. Care Quality Commission, 2019.

[16] Trauer T. Outcome measurement in chronic mental illness. Int Rev Psychiatry 2010; 22(2): 99–113. 20397836 10.3109/09540261003667525

[17] Royal College of Psychiatrists. Health of the Nation Outcomes Scales Guidance for making HoNOS Records. Royal College of Psychiatrists, 2018.

[18] Department of Health. The Mental Health Clustering Booklet v 3.0 2013/14. Department of Health, 2013.

[19] Lenze EJ, Mulsant BH, Blumberger DM, Karp JF, Newcomer JW, Anderson SJ, Efficacy, safety, and tolerability of augmentation pharmacotherapy with aripiprazole for treatment-resistant depression in late life: a randomised, double-blind, placebo-controlled trial. Lancet 2015; 386: 2404–12. Available from: 10.1016/S0140-6736(15)00308-6. 26423182 PMC4690746

[20] Banerjee S, Hellier J, Romeo R, Dewey M, Knapp M, Ballard C, Study of the use of antidepressants for depression in dementia: the HTA-SADD trial – a multicentre, randomised, double-blind, placebo-controlled trial of the clinical effectiveness and cost-effectiveness of sertraline and mirtazapine. Health Technol Assess 2013; 17(7): 1–166. 10.3310/hta17070 PMC4782811 23438937

[21] Singh SP, Greenwood N, White S, Churchill R. Ethnicity and the Mental Health Act 1983. Br J Psychiatry 2007; 191(2): 99–105. 17666492 10.1192/bjp.bp.106.030346

[22] Jacobs R. CHE 48: investigating patient outcome measures in mental health. Care Heal Econ 2009; 48: 1–82.

[23] Jacobs R, Moran V. Uptake of mandatory outcome measures in mental health services. Psychiatrist 2010; 34(8): 338–43.

[24] Parabiaghi A, Rapisarda F, D'Avanzo B, Erlicher A, Lora A, Barbato A. Measuring clinical change in routine mental health care: differences between first time and longer term service users. Aust N Z J Psychiatry 2011; 45(7): 558–68. 21561240 10.3109/00048674.2011.580450

