# Supplementary material for: Effectiveness of treatment for 6813 patients with mental health conditions in Cambridgeshire: a cross-sectional study
Source: BJPsych Open. 2020 Mar 20;6(2):e30. doi: 10.1192/bjo.2020.14 (PMC7176875; doi:10.1192/bjo.2020.14)
Supplement: Supplementary file 1 [file S2056472420000149sup001.docx]

# Supplementary Table 1

***Supplementary Table 1:*** *Coverage of clinical teams that contributed to the CPFT’s HoNOS dataset between 2012 – 2017, broken down by patient diagnostic group and by patient age.*

| **Non-Psychosis** | | | | |
| --- | --- | --- | --- | --- |
| **Service Team Name** | **All Ages** | **<65** | | **65+** |
| Hunt Affective Disorder Pathway | 234 | | 232 | 2 |
| Camb South Affective Disorder Pathway | 73 | | 0 | 73 |
| P'Boro Affective Disorder Pathway | 131 | | 129 | 2 |
| Fenland Affective Disorder Pathway | 155 | | 154 | 1 |
| Camb North Affective Disorder Pathway | 81 | | 1 | 80 |
| 01 NT - Cambridge East | 2 | | 0 | 2 |
| OPMH Huntingdon Locality | 3 | | 0 | 3 |
| OPMH Greater Cambridge Locality | 2 | | 2 | 0 |
| Community Eating Disorders Cambridge | 185 | | 185 | 0 |
| 13 NT - Peterborough City 1 | 2 | | 0 | 2 |
| Personality Disorders Community Service | 111 | | 111 | 0 |
| 10 NT - Isle of Ely | 2 | | 2 | 0 |
| Fenland Perinatal Pathway | 95 | | 95 | 0 |
| 11 NT - Fenland | 2 | | 2 | 0 |
| 02 NT - Cambridge City North | 2 | | 2 | 0 |
| 04 NT - Cambridge North Villages | 2 | | 2 | 0 |
| 12 NT - Wisbech | 2 | | 0 | 2 |
| OPMH East Cambs & Fenland Locality | 2 | | 2 | 0 |
| Community Eating Disorders Norfolk | 118 | | 0 | 118 |
| 09 NT - St Neots | 2 | | 2 | 0 |
| Fenland Assessment Pathway | 38 | | 1 | 37 |
| East Cambs PMHC OP (CLOSED 09/05/16) | 2 | | 0 | 2 |
| CRHT Peterborough | 11 | | 11 | 0 |
| Huntingdon Perinatal Pathway | 18 | | 18 | 0 |
| Camb North Assessment Pathway | 105 | | 0 | 105 |
| OPMH Peterborough & Borderline Locality | 3 | | 3 | 0 |
| CRHTOP North OP | 1 | | 1 | 0 |
| CRHT Cambridge South (CLOSED 19/08/15) | 6 | | 6 | 0 |
| North EDS Community (CLOSED 05/10/15) | 125 | | 0 | 125 |
| Hunt Psychosis Pathway | 30 | | 0 | 30 |
| Central EDS Community (CLOSED 04/10/15) | 41 | | 40 | 1 |
| Camb South Psychosis Pathway | 7 | | 7 | 0 |
| Hunt Assessment Pathway | 66 | | 0 | 66 |
| Cambridge Perinatal Pathway | 17 | | 2 | 15 |
| Peterborough Perinatal Pathway | 10 | | 10 | 0 |
| FACE (CLOSED 06/08/15) | 5 | | 5 | 0 |
| P'Boro Psychosis Pathway | 32 | | 32 | 0 |
| P'Boro Personality Disorder Pathway | 9 | | 8 | 1 |
| 08 NT - St Ives | 1 | | 1 | 0 |
| Camb South Assessment Pathway | 80 | | 80 | 0 |
| CAMEO North | 66 | | 1 | 65 |
| Hunt Personality Disorder Pathway | 8 | | 0 | 8 |
| Fenland Personality Disorder Pathway | 7 | | 7 | 0 |
| 14 NT - Peterborough City 2 | 1 | | 1 | 0 |
| P'Boro Assessment Pathway | 5 | | 0 | 5 |
| Camb North Psychosis Pathway | 4 | | 1 | 3 |
| 03 NT- Camb S Villages (CLOSED 25/07/17) | 2 | | 0 | 2 |
| Hunt Other Pathway | 25 | | 25 | 0 |
| On Call Team | 15 | | 15 | 0 |
| 15 NT - Borderline | 1 | | 1 | 0 |
| Silver Birch Day Thrpy (CLOSED 16/03/16) | 1 | | 1 | 0 |
| Deighton Day Therapy (CLOSED 16/03/16) | 1 | | 1 | 0 |
| CRHT Cambridge | 66 | | 0 | 66 |
| CAMEO South | 16 | | 0 | 16 |
| 07 NT - Huntingdon Central | 3 | | 0 | 3 |
| CRHTOP South OP | 2 | | 2 | 0 |
| PhysiotherapyAdultCambs(CLOSED 23/03/16) | 206 | | 205 | 1 |
| CLASS Team | 10 | | 10 | 0 |
| Oak 3 AAU | 10 | | 9 | 1 |
| Fenland Psychosis Pathway | 4 | | 4 | 0 |
| North Acute Care Serv (CLOSED 06/08/15) | 4 | | 4 | 0 |
| 05 NT - Cambridge City South | 2 | | 2 | 0 |
| 16 NT - Borderline Central | 1 | | 1 | 0 |
| 06 NT - Cambridge City (CLOSED 25/07/17) | 1 | | 0 | 1 |
| S3 Team (CLOSED 05/10/15) | 121 | | 2 | 119 |
| Adult ADHD | 17 | | 17 | 0 |
| Camb South Personality Disorder Pathway | 11 | | 11 | 0 |
| Fenland Other Pathway | 10 | | 10 | 0 |
| Oak 2 (Treatment Unit) (CLOSED 22/12/16) | 8 | | 8 | 0 |
| Fenland EDS Community (CLOSED 05/10/15) | 7 | | 7 | 0 |
| Assertive Outreach Sth (CLOSED 06/08/15) | 7 | | 7 | 0 |
| Saffron Team | 6 | | 0 | 6 |
| Camb South Others Pathway | 5 | | 0 | 5 |
| Camb North Others Pathway | 3 | | 3 | 0 |
| North Inpatients OP (CLOSED 06/08/15) | 1 | | 1 | 0 |
| P'Boro - LPS - OPMH - IP | 1 | | 1 | 0 |
| Hawthorn Day Therapy (CLOSED 16/03/16) | 1 | | 0 | 1 |
| Fen Day Therapy Nth OP (CLOSED 06/06/16) | 1 | | 0 | 1 |
| Arts Therapies OP | 1 | | 0 | 1 |
| South Acute Care Serv (CLOSED 06/08/15) | 42 | | 0 | 42 |
| Gateway Cambridge Nth (CLOSED 05/10/15) | 14 | | 14 | 0 |
| CASUS | 14 | | 13 | 1 |
| Lifeworks | 7 | | 0 | 7 |
| Tenancy Support Peterborough | 6 | | 6 | 0 |
| Gateway Camb South (CLOSED 06/08/15) | 5 | | 5 | 0 |
| Adult Social Care Peterborough | 5 | | 0 | 5 |
| Forensic Community Service South | 4 | | 0 | 4 |
| CUH - LPS - Adults - OP | 3 | | 3 | 0 |
| Arts Therapies Adult | 3 | | 3 | 0 |
| Speech & Language Adult | 3 | | 3 | 0 |
| ARC | 3 | | 0 | 3 |
| CUH - LPS - Adults -  IP | 2 | | 2 | 0 |
| Poplar (PICU) Peterborough | 1 | | 1 | 0 |
| East Cam Mem Assess OP (CLOSED 09/05/16) | 1 | | 1 | 0 |
| New Cottages Day Thpy (CLOSED 16/03/16) | 1 | | 1 | 0 |
| Essex Comm Psychiatry OP | 1 | | 1 | 0 |
| Liaison Addenbrookes OP(CLOSED 31/12/15) | 1 | | 0 | 1 |
| CUH - LPS - OPMH - IP | 1 | | 0 | 1 |
| Maple 2 OP | 1 | | 0 | 1 |
|  |  | |  |  |
| **Psychosis** | | | | |
| **Service Team Name** | **All Ages** | | **<65** | **65+** |
| CAMEO South | 54 | | 54 | 0 |
| CAMEO North | 59 | | 59 | 0 |
| CAMEO North | 54 | | 54 | 0 |
| P'Boro Psychosis Pathway | 12 | | 0 | 12 |
| P'Boro Affective Disorder Pathway | 38 | | 38 | 0 |
| Camb South Affective Disorder Pathway | 16 | | 16 | 0 |
| Hunt Psychosis Pathway | 8 | | 0 | 8 |
| OPMH Huntingdon Locality | 2 | | 0 | 2 |
| 13 NT - Peterborough City 1 | 2 | | 0 | 2 |
| 11 NT - Fenland | 2 | | 0 | 2 |
| Hunt Affective Disorder Pathway | 24 | | 0 | 24 |
| Camb South Psychosis Pathway | 18 | | 0 | 18 |
| Camb North Psychosis Pathway | 16 | | 16 | 0 |
| 10 NT - Isle of Ely | 2 | | 0 | 2 |
| Camb North Affective Disorder Pathway | 42 | | 41 | 1 |
| Fenland Psychosis Pathway | 3 | | 3 | 0 |
| 01 NT - Cambridge East | 1 | | 1 | 0 |
| OPMH Greater Cambridge Locality | 1 | | 0 | 1 |
| CRHT Cambridge South (CLOSED 19/08/15) | 3 | | 3 | 0 |
| OPMH Peterborough & Borderline Locality | 1 | | 1 | 0 |
| 05 NT - Cambridge City South | 1 | | 1 | 0 |
| Fenland Assessment Pathway | 34 | | 32 | 2 |
| North Acute Care Serv (CLOSED 06/08/15) | 4 | | 0 | 4 |
| CRHTOP North OP | 1 | | 1 | 0 |
| 07 NT - Huntingdon Central | 1 | | 0 | 1 |
| CRHT Peterborough | 18 | | 0 | 18 |
| Camb North Assessment Pathway | 9 | | 0 | 9 |
| Fenland Affective Disorder Pathway | 2 | | 2 | 0 |
| Personality Disorders Community Service | 24 | | 23 | 1 |
| Mulberry 2 | 11 | | 11 | 0 |
| Camb South Assessment Pathway | 5 | | 5 | 0 |
| Tenancy Support Peterborough | 5 | | 0 | 5 |
| Cambridge Perinatal Pathway | 4 | | 4 | 0 |
| South Acute Care Serv (CLOSED 06/08/15) | 2 | | 2 | 0 |
| Oak 1 (Treatment Unit) | 2 | | 2 | 0 |
| 14 NT - Peterborough City 2 | 2 | | 0 | 2 |
| 06 NT - Cambridge City (CLOSED 25/07/17) | 1 | | 1 | 0 |
| 02 NT - Cambridge City North | 1 | | 1 | 0 |
| 09 NT - St Neots | 1 | | 1 | 0 |
| OPMH East Cambs & Fenland Locality | 1 | | 0 | 1 |
| 12 NT - Wisbech | 1 | | 0 | 1 |
| Hunt Assessment Pathway | 16 | | 15 | 1 |
| George Mackenzie | 11 | | 11 | 0 |
| Clozapine Clinic | 5 | | 0 | 5 |
| IOM (CLOSED 07/03/16) | 4 | | 4 | 0 |
| Arts Therapies Adult | 4 | | 0 | 4 |
| CLASS Team | 3 | | 3 | 0 |
| On Call Team | 2 | | 2 | 0 |
| Hunt Other Pathway | 2 | | 2 | 0 |
| Oak 2 (Treatment Unit) (CLOSED 22/12/16) | 2 | | 2 | 0 |
| 15 NT - Borderline | 2 | | 2 | 0 |
| Maple 1 OP | 2 | | 0 | 2 |
| Assertive Outreach Sth (CLOSED 06/08/15) | 1 | | 1 | 0 |
| Camb South Others Pathway | 1 | | 1 | 0 |
| Willow OP | 1 | | 0 | 1 |
| CRHTOP South OP | 1 | | 0 | 1 |
| 08 NT - St Ives | 1 | | 0 | 1 |
| 03 NT- Camb S Villages (CLOSED 25/07/17) | 1 | | 0 | 1 |
| North Inpatients OP (CLOSED 06/08/15) | 1 | | 0 | 1 |
| CUH - LPS - OPMH - IP | 1 | | 0 | 1 |
|  |  | |  |  |
| **Organic** | | | | |
| **Service Team Name** | **All Ages** | | **<65** | **65+** |
| OPMH Peterborough & Borderline Locality | 258 | | 4 | 254 |
| OPMH Huntingdon Locality | 164 | | 3 | 161 |
| OPMH Greater Cambridge Locality | 18 | | 0 | 18 |
| OPMH East Cambs & Fenland Locality | 78 | | 2 | 76 |
| 01 NT - Cambridge East | 17 | | 16 | 1 |
| 10 NT - Isle of Ely | 25 | | 0 | 25 |
| 03 NT- Camb S Villages (CLOSED 25/07/17) | 8 | | 0 | 8 |
| 07 NT - Huntingdon Central | 21 | | 0 | 21 |
| 11 NT - Fenland | 21 | | 0 | 21 |
| 06 NT - Cambridge City (CLOSED 25/07/17) | 3 | | 0 | 3 |
| 13 NT - Peterborough City 1 | 996 | | 74 | 922 |
| 13 NT - Peterborough City 1 | 16 | | 16 | 0 |
| 02 NT - Cambridge City North | 14 | | 1 | 13 |
| 04 NT - Cambridge North Villages | 2 | | 0 | 2 |
| 09 NT - St Neots | 8 | | 0 | 8 |
| CRHTOP North OP | 5 | | 0 | 5 |
| 12 NT - Wisbech | 7 | | 0 | 7 |
| YPwD South OP | 78 | | 2 | 76 |
| 16 NT - Borderline Central | 17 | | 2 | 15 |
| YPwD North OP | 191 | | 2 | 189 |
| East Cam Mem Assess OP (CLOSED 09/05/16) | 1 | | 1 | 0 |
| 14 NT - Peterborough City 2 | 121 | | 0 | 121 |
| 08 NT - St Ives | 2 | | 2 | 0 |
| 15 NT - Borderline | 1 | | 1 | 0 |
| East Cambs PMHC OP (CLOSED 09/05/16) | 1 | | 1 | 0 |
| Fen Day Therapy Nth OP (CLOSED 06/06/16) | 11 | | 0 | 11 |
| Denbigh OP | 2 | | 0 | 2 |
| Hawthorn Day Therapy (CLOSED 16/03/16) | 13 | | 0 | 13 |
| CRHTOP South OP | 2 | | 2 | 0 |
| Essex Comm Psychiatry OP | 2 | | 0 | 2 |
| P'Boro Affective Disorder Pathway | 562 | | 8 | 554 |
| Hunt Affective Disorder Pathway | 454 | | 3 | 451 |
| Deighton Day Therapy (CLOSED 16/03/16) | 1 | | 0 | 1 |
| New Cottages Day Thpy (CLOSED 16/03/16) | 1 | | 0 | 1 |
| Liaison Addenbrookes OP(CLOSED 31/12/15) | 1 | | 0 | 1 |
| FACE (CLOSED 06/08/15) | 254 | | 1 | 253 |
| Fenland Assessment Pathway | 176 | | 0 | 176 |
| Camb South Affective Disorder Pathway | 149 | | 4 | 145 |
| YPwD North Reviews | 68 | | 1 | 67 |
| Camb North Assessment Pathway | 16 | | 0 | 16 |
| CUH - LPS - Adults - OP | 13 | | 0 | 13 |
| Older People Social Care Peterborough | 1 | | 1 | 0 |
| Speech & Language OP | 1 | | 1 | 0 |
| Silver Birch Day Thrpy (CLOSED 16/03/16) | 1 | | 0 | 1 |
| CUH - LPS - OPMH - IP | 1 | | 0 | 1 |
| Physiotherapy North OP (CLOSED 06/06/16) | 1 | | 0 | 1 |
| 05 NT - Cambridge City South | 1 | | 0 | 1 |
